# Restoration of Normal Brain Connectivity With Deep Brain Stimulation for Drug-Resistant Epilepsy in a Pediatric Patient

**DOI:** 10.7759/cureus.98048

**Published:** 2025-11-28

**Authors:** Isabel Martin del Campo, Bayron A Sandoval-Bonilla

**Affiliations:** 1 Neurosurgery, Anáhuac University, Mexico City, MEX; 2 Neurosurgery, Mexican Social Security Institute, Mexico City, MEX; 3 National System of Researchers (Sistema Nacional de Investigadores, SNI), National Council of Humanities, Sciences, and Technologies, Mexico City, MEX

**Keywords:** deep brain stimulation, default mode network, drug-resistant epilepsy, neuromodulation, resting-state

## Abstract

Epilepsy is recognized as a network disorder. For drug-resistant epilepsy (DRE) that is not amenable to focal resection, deep brain stimulation (DBS) can modulate large-scale networks and improve seizure control.

A 16‑year‑old patient with long‑standing DRE underwent hippocampal DBS. Following posterior lead migration, the patient developed a sustained clinical benefit, with seizure frequency decreasing from 12 to 16 daily events to occasional, non-disabling episodes occurring less than once per month - an evolution from Engel Class IVA to Engel Class IC in the Engel Epilepsy Surgery Outcome Classification. After a generator replacement in 2019, resting‑state functional magnetic resonance imaging (rs‑fMRI) was acquired with the stimulator off at discharge, and subsequently at one week and one month, and compared with a healthy control.

The rs‑fMRI off‑stimulation imaging showed noticeable hypoconnectivity within the default mode network (DMN). At one week of stimulation, DMN connectivity remained reduced. One month after reactivation, DMN connectivity resembled the control pattern, paralleling the improvement in seizure control.

This single‑case observation suggests that DBS may reversibly modulate intrinsic brain networks in DRE, with a time-dependent restoration of DMN connectivity. Although preliminary and exploratory in nature, these findings motivate prospective studies to confirm mechanisms and the durability of network normalization with DBS.

## Introduction

Drug-resistant epilepsy (DRE) is a therapeutic challenge because failure of antiseizure medications profoundly affects the quality of life and cognitive development of patients [1]. Epilepsy is a network disease. In focal-onset epilepsies, resection of the epileptogenic zone interrupts pathological circuits, restoring functional connectivity within brain networks and improving seizure control [2]. Patients with multifocal or generalized-onset epilepsies are usually not candidates for focused surgical resection and may benefit from neuromodulation, which provides a reversible disruption of the epileptic network [1].

We report a case illustrating the neuromodulation of large-scale brain connectivity through deep brain stimulation (DBS). Using resting-state functional MRI (rs-fMRI), we observed changes in the default mode network (DMN) after stimulation, suggesting network-level effects beyond local target activation.

## Case presentation

A 16-year-old patient with a nine-year history of DRE completed a comprehensive presurgical evaluation consistent with left temporal lobe epilepsy (TLE). The family declined resective surgery, and a left hippocampal depth electrode was implanted for DBS. Approximately one month later, imaging showed posterior lead migration. This finding coincided with a substantial decrease in seizure frequency, and stimulation was initiated. Seizure control improved from 12 to 16 daily seizures to a sustained occasional, non-disabling event (less than one per month) - an evolution from preoperative Engel Class IVA (Engel Epilepsy Surgery Outcome Classification) to Engel Class IC at the most recent follow-up. Programming started at low amplitudes and was titrated over time; stimulation settings across visits (voltage/current, pulse width, frequency, impedance) are summarized in Table 1 (typical settings: 2.0-4.0 V, 120-150 µs, 145 Hz).

**Table 1:** Stimulation parameters of posterior Sylvian junction DBS. * Because the battery level was 2.31 V in an Elective Replacement Indicator (ERI) mode, impedance lecture was not performed. DBS: deep brain stimulation Credit: Table reproduced with permission from [3].

Programming	V	us	Hz	Ohms	mA	C	0	1	2	3	Seizure frequency
Preoperative											12-16 daily
Postoperative week 1-3	Hippocampal lead										12-16 daily
Postoperative week 4	Lead migration										3-4 daily seizures
Initial	2.0	120	145	492	3.933	+	-	-	-	-	Engel IC
05/2019	2.0	120	145	492	3.933	+	-	-	-	-	
08/2019	2.2	120	145	927	2.4	+			-	-	
12/2019	2.2	120	145	927	2.4	+	-	-			
09/2020	2.0	120	145	611	3.226	+	-	-	-	-	
12/2020	3.0	120	145	611	4.8	+	-	-	-	-	
05/2021	4.0	120	145	*	*	+	-	-	-	-	
08/2021	4.0	150	145	527	7.4	+	-	-	-	-	
12/2021	4.0	150	145	488	8.0	+	-			-	
Current	4.0	150	145	488	8.0	+	-	-	-	-	

In 2019, during elective generator replacement, rs-fMRI was performed OFF-stimulation at discharge, then ON at one week and ON at one month, to explore network-level effects.

After elective generator replacement, programming changes were planned. The stimulator was not activated post-surgery immediately. An initial rs-fMRI was therefore obtained with stimulation OFF. To analyze the DMN, we applied seed-based correlation and graph-theory metrics as independent approaches.

The patient was discharged the next day, and the DBS was reactivated using previously effective parameters (4.0 V, 150 µs, 145 Hz; contacts 0-3 negative, case positive). A second rs-fMRI was acquired one week after discharge (ON-stimulation). A third rs-fMRI was obtained one month after stimulation had been restored; the same parameters were maintained.

**Figure 1:**
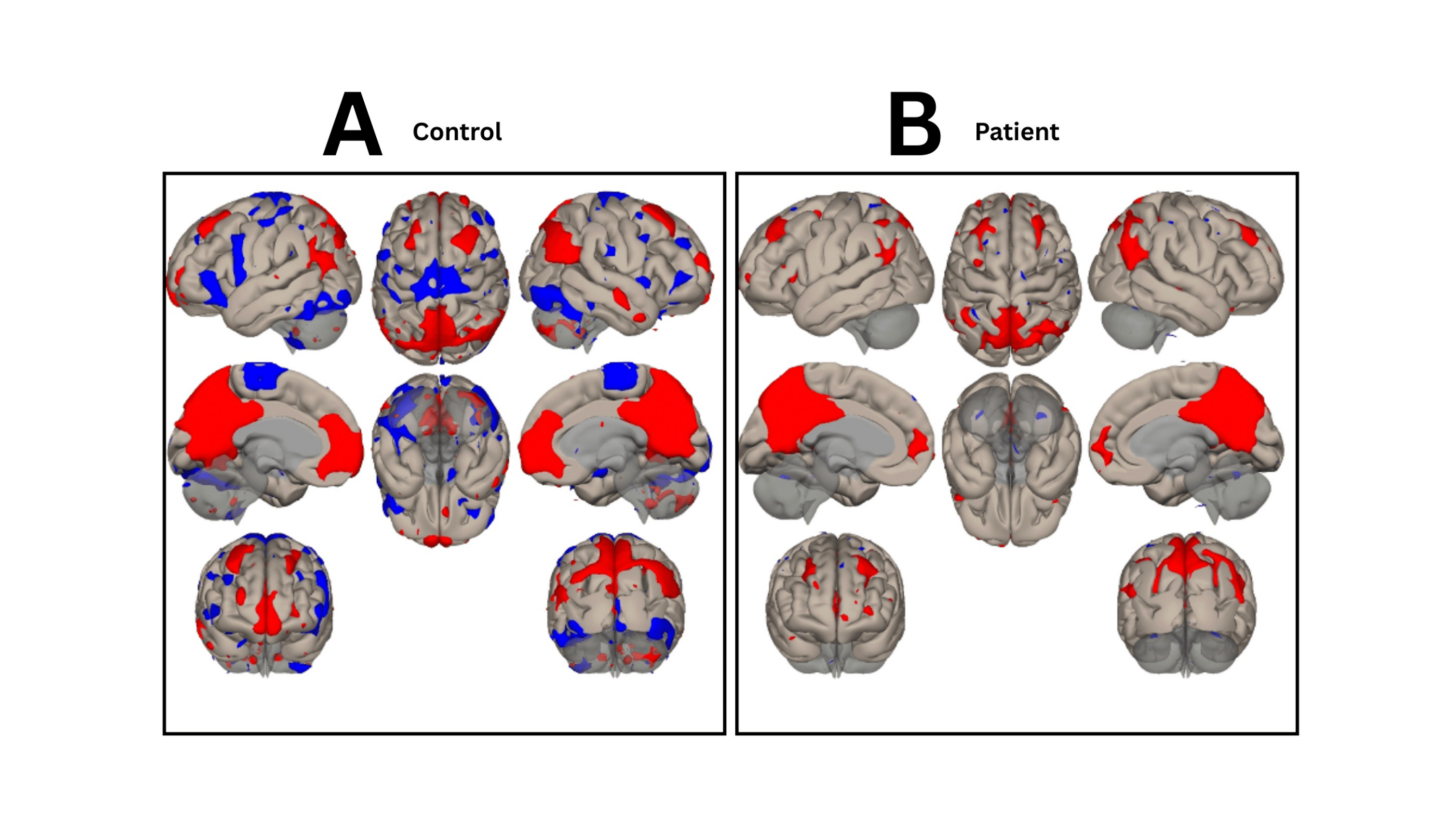
Resting-state fMRI in (A) a healthy control and (B) the patient before DBS re-activation (OFF-stimulation at discharge), showing altered network connectivity within the patient’s epileptic network. fMRI: functional magnetic resonance imaging; DBS: deep brain stimulation

For comparison, we also acquired rs-fMRI from a healthy control without epilepsy or neurological disease, age-matched to the patient (Figure 1).

**Figure 2:**
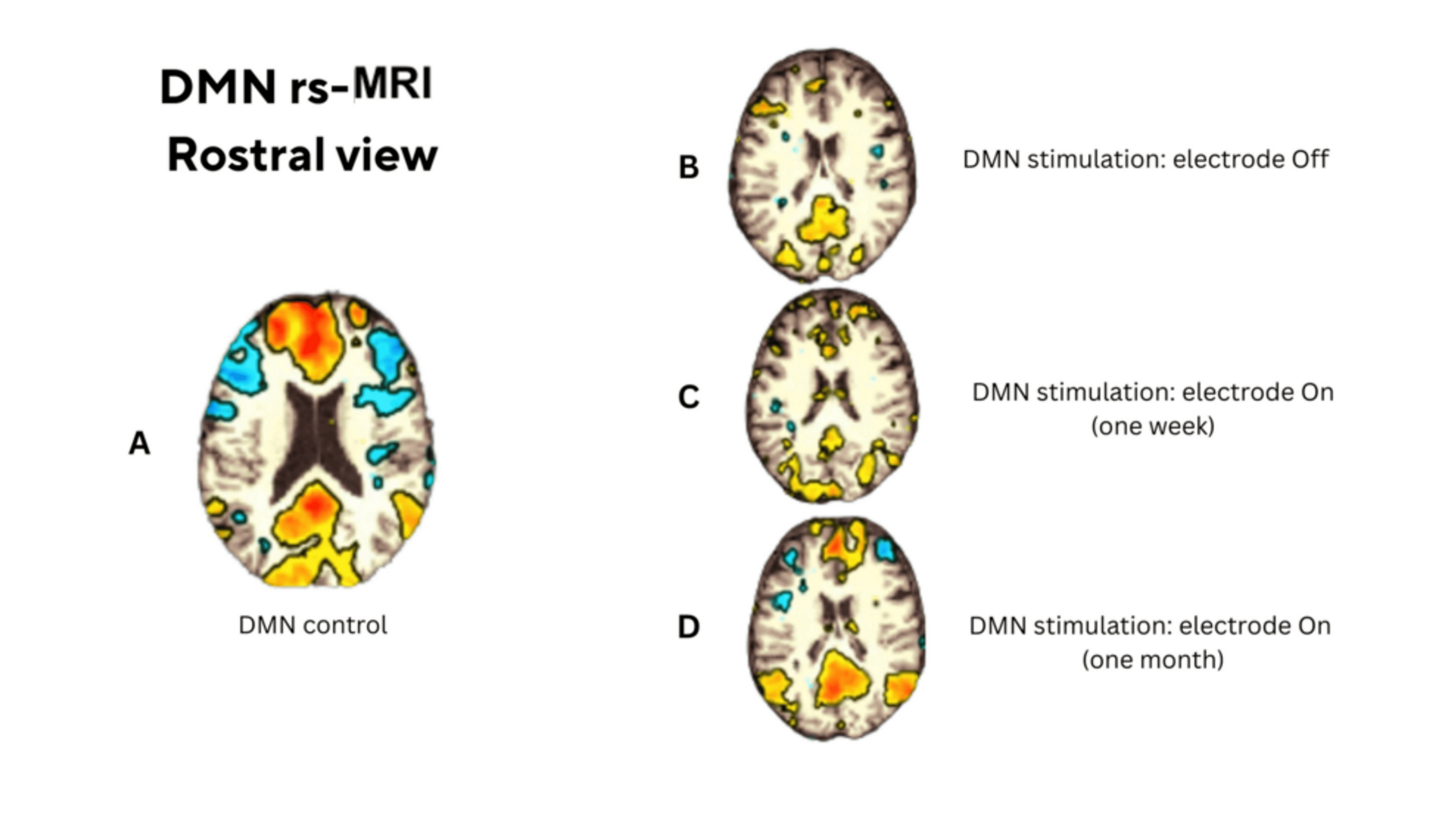
Rostral views of rs-fMRI DMN maps. (A) Healthy control. (B) Patient OFF-stimulation at discharge. (C) Patient ON-stimulation at one week. (D) Patient ON-stimulation at one month, demonstrating a DMN configuration similar to that of the control. rs-fMRI: resting‑state functional magnetic resonance imaging; DMN: default mode network

**Figure 3:**
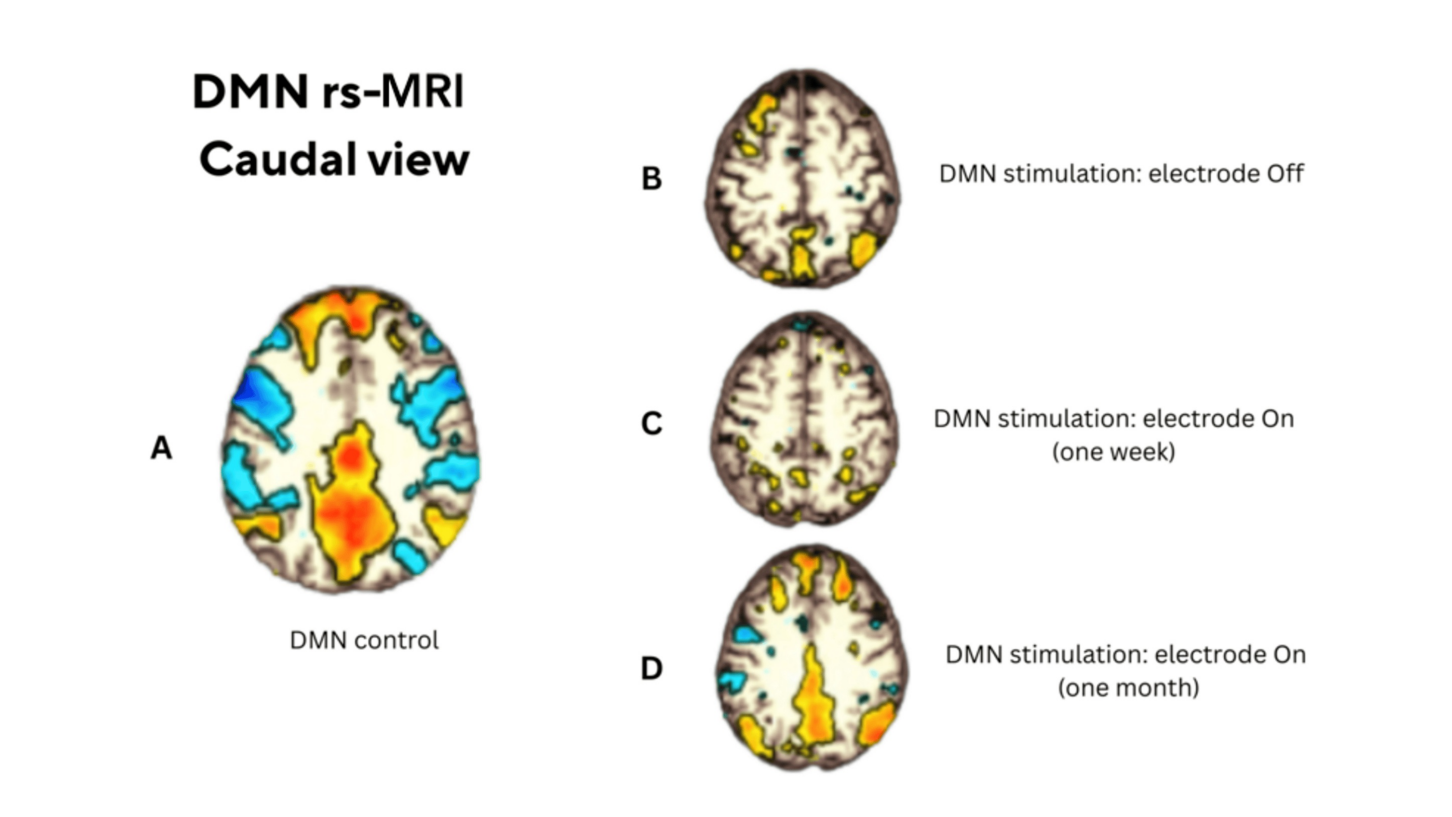
Caudal views of rs-fMRI DMN maps. (A) Healthy control. (B) Patient OFF-stimulation. (C) Patient ON-stimulation at one week. (D) Patient ON-stimulation at one month, illustrating progressive recovery of DMN connectivity over time following stimulation. rs-fMRI: resting‑state functional magnetic resonance imaging; DMN: default mode network

At hospital discharge (OFF-stimulation), rs-fMRI showed markedly reduced functional connectivity within the DMN. One week after reactivation (ON-stimulation), DMN connectivity had not yet recovered. By one month after stimulation had been restored, rs-fMRI demonstrated a DMN pattern resembling that of the healthy control, consistent with restoration of large-scale network connectivity (Figures 2-3).

## Discussion

The DMN is the prototypical task-negative network identified with rs-fMRI. During wakeful rest, several large-scale networks can be delineated in the human cortex, reflecting intrinsic low-frequency BOLD fluctuations rather than externally driven activity [4]. Canonical networks include the DMN, visual, sensorimotor, auditory, dorsal/ventral attention, salience, executive-control, reward/emotion, and language, with variable degrees of spatial overlap and anti-correlation among them [5]. Functional connectivity among regions of interest at rest can be quantified with multiple analytic approaches, such as seed-based correlation and graph-theory metrics, which we used independently in this report [6].

**Figure 4:**
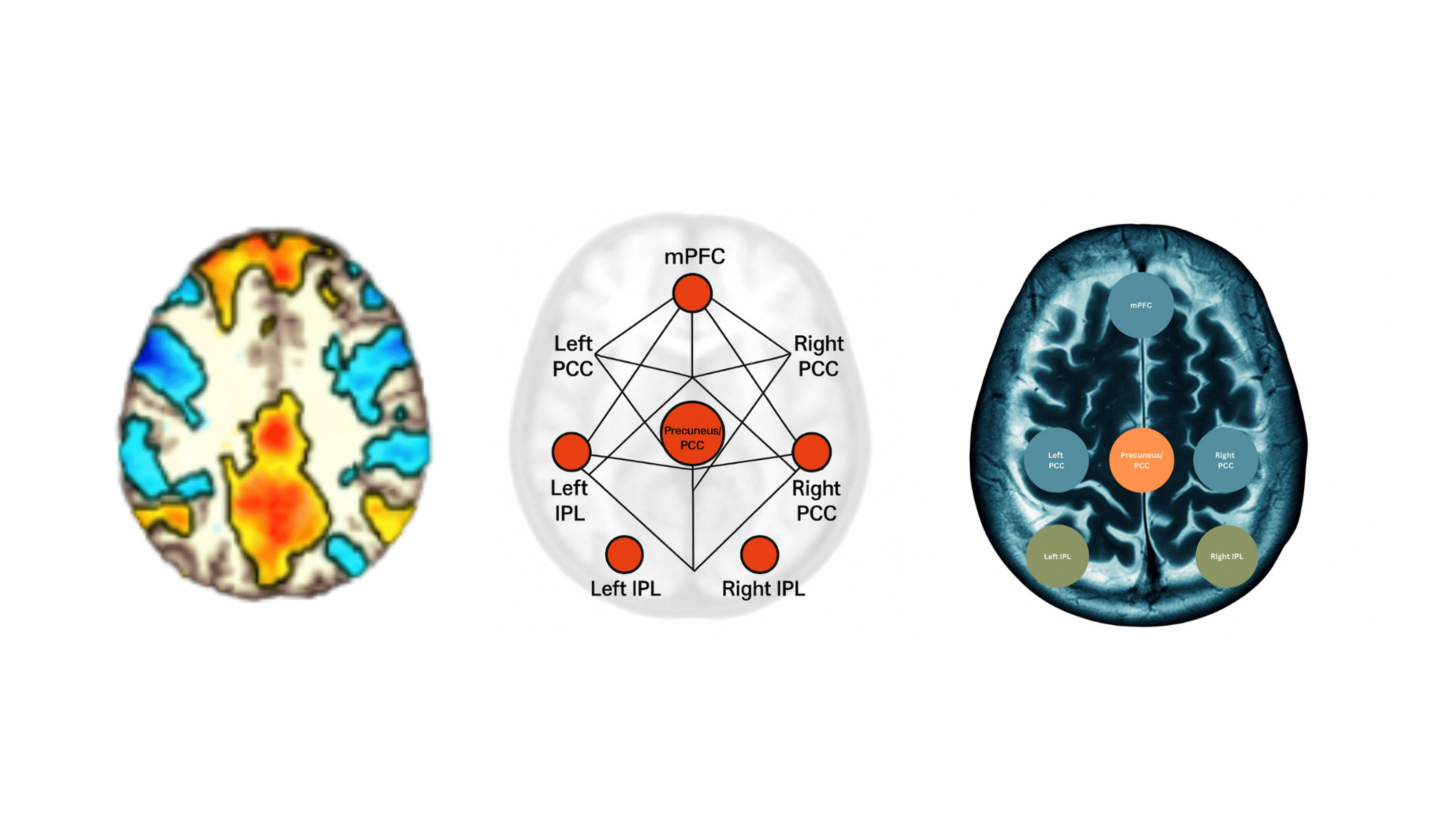
Schematic of default mode network (DMN) cortical hubs, including the medial prefrontal cortex (mPFC), posterior cingulate cortex/precuneus (PCC/Prec), and bilateral inferior parietal lobules (IPL); mesial and lateral temporal regions, including the hippocampus, also contribute.

Anatomically, the DMN is anchored in the medial prefrontal cortex (mPFC) and the posterior cingulate cortex/precuneus (PCC/Prec), with prominent hubs in the bilateral inferior parietal lobules (IPL); mesial and lateral temporal regions, including hippocampal structures, also contribute to the network (Figure 4) [7]. Disruption of connectivity within these nodes has been associated with cognitive and clinical manifestations across neurologic disorders, including epilepsy.

TLE is the most common focal epilepsy, and alterations of DMN connectivity have been consistently reported. Maccotta et al. studied 17 patients with drug-resistant TLE who underwent anterior temporal lobectomy or selective amygdalohippocampectomy, comparing rs-fMRI before and after surgery with 17 matched controls. Patients showed reduced DMN connectivity relative to controls both pre- and post-operatively, despite achieving Engel Class I seizure control in all cases [8]. These findings suggest that, at the time scale of early postoperative imaging and after long disease duration, network abnormalities may not immediately normalize. Other reports indicate that seizure freedom in TLE can involve hub redistribution within the DMN, and that lower connectivity/slower system responsiveness may limit propagation of epileptic discharges after surgery. Importantly, these results derive from heterogeneous etiologies (e.g., tumors, hippocampal sclerosis, focal cortical dysplasia), which may drive etiology-specific network reorganization [7].

In TLE, the dorsal DMN appears more affected than the ventral component both before and after surgery, indicating non-uniform vulnerability of DMN subsystems [9]. Compensatory neuroplasticity may thus depend on the subnetwork location of focal-onset epilepsy. Connectivity studies centered on the cingulate-hippocampal axis show reduced coupling between the PCC and the epileptogenic hippocampus preoperatively, with increased contralateral PCC-hippocampus connectivity. After resection, contralateral PCC-hippocampal coupling further increases and has been associated with postoperative memory outcome [10].

In idiopathic generalized epilepsies (IGE), functional interactions among DMN hubs have also been demonstrated, and refractory epilepsy has been linked to reduced connectivity within these hubs [11,12]. Resective surgery induces connectome reorganization with heterogeneous patterns across etiologies; several studies have documented DMN differences when comparing focal epilepsies before versus after removal of the epileptogenic zone [2]. Accordingly, rs-fMRI-derived DMN maps have been proposed as biomarkers for presurgical evaluation and postoperative follow-up in epilepsy surgery [13,14].

For patients with DRE who are not candidates for, or decline, resection, DBS is a valuable therapeutic option. Evidence supports DBS for seizure reduction, particularly stimulation of the anterior nucleus of the thalamus (ANT) [15,16]. The prevailing view is that ANT-DBS modulates thalamo-cortical circuits, stabilizing hyperexcitable networks. Other targets have also shown benefit in selected populations, including the centromedian thalamic nucleus (CM) in generalized epilepsies and the hippocampus TLE [1].

Our case illustrates that DBS can be associated with substantial seizure reduction, together with time-dependent changes on serial rs-fMRI. When the generator was OFF, DMN connectivity of the patient was reduced compared with the non-epileptic control; one month after re-activation, the DMN approximated the control pattern. These observations suggest that neuromodulation may gradually reconfigure large-scale networks in parallel with clinical improvement. While causality cannot be established in a single case, the combined clinical response and DMN evolution are consistent with network-level effects described for DBS in DRE [17].

Pediatric epilepsy poses unique challenges due to ongoing brain development and psychosocial factors. Evidence supports the feasibility and effectiveness of DBS in children with DRE who are not candidates for, or who decline, resection, with meaningful seizure reductions reported across series [18]. Conceptually, connectivity hubs/confluence points may represent complementary targets for neuromodulation in network-level epilepsies [3]. Moreover, long-term cohorts indicate that DBS can show sustained benefit in seizure control and patient-reported outcomes, underscoring its therapeutic durability in appropriately selected cases [19,20].

## Conclusions

Epilepsy is a disorder of large-scale brain networks, and rs-fMRI consistently shows network alterations in affected patients. In this pediatric case of DRE, DBS was associated with a marked clinical improvement and a time-dependent restoration of DMN connectivity, suggesting that some network abnormalities may be functionally reversible with neuromodulation.

This observation derives from a single case and cannot establish causality. Factors such as epilepsy duration, etiology, comorbidities, medication changes, and programming parameters may influence connectivity. Therefore, these findings should be considered preliminary and require confirmation in larger, prospective studies to clarify mechanisms, durability, and clinical utility of rs-fMRI as a biomarker of DBS effects.
